# Re: Is cancer back? Psychological issues faced by survivors of breast cancer

**DOI:** 10.1590/1806-9282.20250628

**Published:** 2026-01-09

**Authors:** Ilker Sengul, Demet Sengul

**Affiliations:** 1 Giresun University, Faculty of Medicine, Division of Endocrine Surgery – Giresun, Turkey.; 2 Giresun University, Faculty of Medicine, Department of General Surgery – Giresun, Turkey.; 3 Giresun University, Faculty of Medicine, Department of Pathology – Giresun, Turkey.

Dear Editor,

*Emendatio pars studiorum longe utilissima. A Deucalione*, mastology remains an important issue in human beings^1-8^. We have read with great interest the article "Is cancer back?—psychological issues faced by survivors of breast cancer"^9^. Bergerot and colleagues^9^ purposed to investigate the multifaceted sentimental complexities faced by survivors, incorporating a variety of issues from fear of recurrence to body image distrustfulness, which underscores the compulsory support. The authors reviewed the information through the PubMed index with personal files and examined the risk factors contributing to augmented psychological distress with periods of sentimentality. Of note is that they negotiate and discourse psychological interventions to upgrade resilience and develop the quality of life of survivors. In addition, they also stressed the requirement for ongoing research on survival into the long-term psychological effects of cancer recurrence. Nevertheless, would more extensive sampling for the clinical studies, which might possibly eliminate bias, alter this valued work's outcome(s)? Moreover, would it be convenient and suitable to emphasize the overt criteria for the article selection and demographic diversity of survivors studied for this work? Does a deficiency of quantitative data or meta-analyses, which might hinder the adroitness of comprehending the emotional confusion of the survivors, limit the tendency to generalize the relevant findings? This issue merits further investigation. We thank Bergerot et al.^9^ for their study on breast cancer survivors.

## Data Availability

The datasets generated and/or analyzed during the current study are available from the corresponding author upon reasonable request.
